# Molecular Mechanism of Naringenin Against High-Glucose-Induced Vascular Smooth Muscle Cells Proliferation and Migration Based on Network Pharmacology and Transcriptomic Analyses

**DOI:** 10.3389/fphar.2022.862709

**Published:** 2022-06-09

**Authors:** Wenjun He, Yanming Wang, Rui Yang, Huihui Ma, Xuqing Qin, Meijuan Yan, Yi Rong, Yufang Xie, Li Li, Junqiang Si, Xinzhi Li, Ketao Ma

**Affiliations:** ^1^ Key Laboratory of Xinjiang Endemic and Ethnic Diseases, Ministry of Education, Shihezi University School of Medicine, Shihezi, China; ^2^ NHC Key Laboratory of Prevention and Treatment of Central Asia High Incidence Diseases, First Affiliated Hospital, Shihezi University School of Medicine, Shihezi, China; ^3^ Department of Pathophysiology, Shihezi University School of Medicine, Shihezi, China; ^4^ Department of Physiology, Shihezi University School of Medicine, Shihezi, China

**Keywords:** diabetic angiopathies, naringenin, network pharmacology, transcriptomics, molecular docking

## Abstract

Although the protective effects of naringenin (Nar) on vascular smooth muscle cells (VSMCs) have been confirmed, whether it has anti-proliferation and anti-migration effects in high-glucose-induced VSMCs has remained unclear. This study aimed to clarify the potential targets and molecular mechanism of Nar when used to treat high-glucose-induced vasculopathy based on transcriptomics, network pharmacology, molecular docking, and *in vivo* and *in vitro* assays. We found that Nar has visible anti-proliferation and anti-migration effects both *in vitro* (high-glucose-induced VSMC proliferation and migration model) and *in vivo* (type 1 diabetes mouse model). Based on the results of network pharmacology and molecular docking, vascular endothelial growth factor A (VEGFA), the proto-oncogene tyrosine-protein kinase Src (Src) and the kinase insert domain receptor (KDR) are the core targets of Nar when used to treat diabetic angiopathies, according to the degree value and the docking score of the three core genes. Interestingly, not only the Biological Process (BP), Molecular Function (MF), and KEGG enrichment results from network pharmacology analysis but also transcriptomics showed that phosphatidylinositol-3-kinase (PI3K)/protein kinase B (Akt) is the most likely downstream pathway involved in the protective effects of Nar on VSMCs. Notably, according to the differentially expressed genes (DEGs) in the transcriptomic analysis, we found that cAMP-responsive element binding protein 5 (CREB5) is a downstream protein of the PI3K/Akt pathway that participates in VSMCs proliferation and migration. Furthermore, the results of molecular experiments *in vitro* were consistent with the bioinformatic analysis. Nar significantly inhibited the protein expression of the core targets (VEGFA, Src and KDR) and downregulated the PI3K/Akt/CREB5 pathway. Our results indicated that Nar exerted anti-proliferation and anti-migration effects on high-glucose-induced VSMCs through decreasing expression of the target protein VEGFA, and then downregulating the PI3K/Akt/CREB5 pathway, suggesting its potential for treating diabetic angiopathies.

## Graphical Abstract

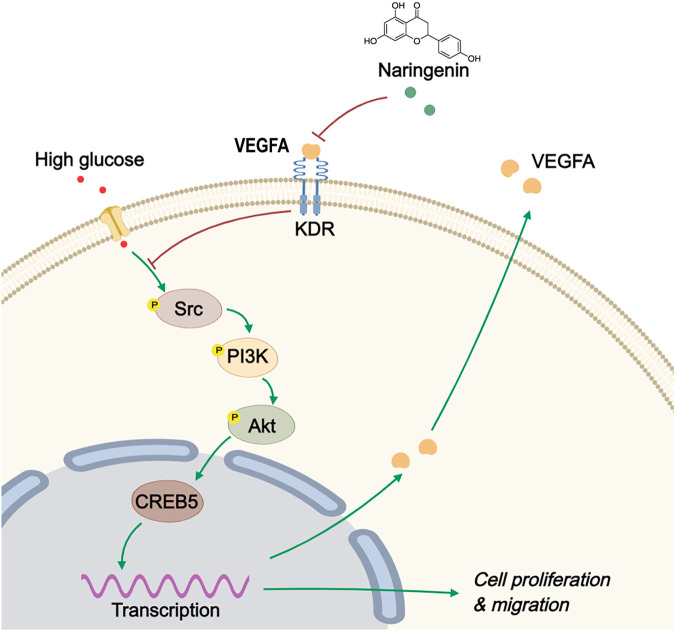

## 1 Introduction

The morbidity of diabetes characterized by abnormally elevated glucose is increasing year by year across the world, making it one of the most serious burdens on healthcare resources globally (Forbes and Cooper, 2013). The diabetic complications that arise with the progression of diabetes are major causes of low quality of life and high mortality among patients, which include retinopathy, nephropathy, neuropathy, myocardial infarction, and cerebrovascular disease (Deshpande et al., 2008). In particular, cardiovascular diseases (CVDs) due to microvascular and macrovascular lesions are major causes of death in diabetics. The anomalous blood glucose level is closely related to such vascular lesions. Under normal physiological conditions, vascular smooth muscle cells (VSMCs) tend to accumulate at the G0/G1 phase of the cell cycle, which is characterized by low proliferation, and an ordered, differentiated, and contractile phenotype for achieving contractile and synthetic function (Bruemmer and Law, 2003). However, under pathological conditions, VSMCs have a more pronounced ability to proliferate and migrate (Jiang et al., 2017). Numerous studies have reported that chronic hyperglycemia is directly responsible for the initiation and exacerbation of a transition in vascular phenotype from the normal, stabilized contractile phenotype to the synthetic phenotype with the development and progression of diabetes (Shi et al., 2020). Additionally, the excessive proliferation of VSMCs can lead to the development and exacerbation of hypertension, atherosclerosis, and restenosis (Ran et al., 2021). Therefore, modification of the VSMC phenotype is a potential treatment strategy for various vascular diseases in diabetic patients.

Naringenin (Nar) (PubChem CID: 932) is a member of the flavonoids obtained from citrus fruits, including grapefruit, orange, and tomato (Arafah et al., 2020). Evidence has shown that it has various pharmacological activities, including anti-inflammatory, anti-tumor, anti-bacterial, anti-diabetic, anti-oxidant, immunomodulatory, hepatoprotective, and cardiovascular protective effects (Den Hartogh and Tsiani, 2019). Salehi et al. reported a clinical trial that showed that, through long-term daily supplementation with grapefruit (containing naringenin glycoside), the endothelial function of 48 healthy menopausal women was improved (Salehi et al., 2019). In addition, Chen et al. reported that Nar can inhibit VSMC proliferation and migration by upregulating HO-1 expression in a TNF-α-induced VSMC proliferation model (Chen et al., 2012). Moreover, Lee et al. found that both Nar and naringin had protective effects on the cardiovascular system by inhibiting MMP expression and Akt activity in a TNF-α-induced VSMC proliferation model (Lee et al., 2009). These studies indicate that Nar has potential beneficial effects on VSMCs. However, whether Nar has beneficial effects on high-glucose-induced VSMC proliferation and migration remains unclear. Therefore, in the present study, we further explored the effect of Nar on VSMC proliferation and migration induced by high glucose, and examined the possible molecular mechanisms involved by combining network pharmacology and transcriptomics. Our findings demonstrated that Nar could attenuate the high-glucose-induced proliferation and migration of VSMCs through inhibiting VEGFA target expression and downregulating the PI3K/Akt/CREB5 signaling pathway, which indicated Nar’s potential for treating vascular lesions in diabetic vessels.

## 2 Materials and Methods

### 2.1 Materials

Naringenin (Nar) (≥ 95%, N5893), streptozotocin (STZ) were purchased from Sigma (Sigma Aldrich, United States). Anti-PCNA (1:1000, ab29) and anti-OPN (1:500, ab63856) were purchased from Abcam. Anti-MMP2 (1:1000, bs-4605R) and anti-MMP9 (1:1000, bsm-54040R) were obtained from Bioss Biotechnology Co., LTD. (Beijing, China). Anti-α-SMA (1:1000, BM0002), anti-VEGFA (1:1000, BA0407) and anti-VEGFR2/KDR (1:1000, A00901-3) were purchased from Boster Biological Technology Co. Ltd. (Shanghai, China). Anti-CREB5 (1:1000, G420) was purchased from Santa cruz. The following antibodies: PI3K (1:1000, 4249), p-PI3K (1:500, 17366), Akt-pan (1:1000, 4685), p-Akt (Ser 473) (1:1000, 4060), Src (1:1000, 36D10), and p-Src (1:1000, D49G4) were obtained from CST. EdU Imaging kit was purchased from APE × Bio (United States). Trypsin, Dulbecco’s Modified Eagle’s Medium (DMEM) and fetal bovine serum (FBS) were purchased from GIBCO.

### 2.2 Animals and Model Preparation

Male C57BL/6 mice aged 6–8 weeks (20 ± 2 g) were obtained from Hengzhao Biotechnology Co., Ltd. All animal experiments were performed in accordance with standard animal research guidelines. Mice were housed under a 12 h light/12 h dark cycle in a temperature-controlled room, and maintained on standard chow and tap water ad libitum.

To establish the diabetic model, 48 mice were randomly assigned to two groups. The Control group contained 12 mice and the diabetic group 36. After fasting for 12 h, mice in the diabetic group were intraperitoneally injected with streptozotocin (STZ, Sigma) (40 mg/kg body wt.) dissolved in sodium citrate buffer (pH 4.2) for 5 consecutive days (Nikolic et al., 2014). Control mice were injected with an equal volume of buffer solution. Three days after injection with STZ, mice with non-fasting blood glucose levels > 16.6 mmol/L were considered to have been successfully induced to develop diabetes and used for subsequent experiments.

Thirty-six diabetic mice were randomly divided into three groups: a model group (DM), a low-dose Nar group (DM + LN) (25 mg/kg), and a high-dose Nar group (DM + HN) (75 mg/kg) (Zhang et al., 2018). Nar was dissolved in saline (Wang et al., 2016), and intragastrically administered once a day for 12 weeks. The model and control mice were intragastrically administered an equal volume of saline. After 12 weeks of treatment, all mice were euthanized, and the thoracic aorta was isolated and used for subsequent experiments.

### 2.3 Cell Culture and Treatments

The primary Sprague-Dawley rat VSMCs were isolated from the thoracic aorta of male rats by the tissue adherence method, and characterized by smooth muscle-specific α-actin staining, as described previously (Sun et al., 2016). Only cells at passages 3-6 were used for the *in vitro* experiment. The VSMCs were cultured in DMEM (normal glucose) containing 10% FBS and 1% antibiotic-antimycotic (containing penicillin, streptomycin, and amphotericin B) at 37°C in a 5% CO2 and 95% air incubator, as described previously (Hashim et al., 2006).

### 2.4 Hematoxylin–Eosin Staining

The thoracic aorta was isolated and fixed in 4% paraformaldehyde, embedded in paraffin, and transversely cut into 5 μm sections with a cryostat. The sections were subjected to hematoxylin–eosin (HE) staining (Fan et al., 2012) and then imaged with a light microscope. Media thickness, lumen diameter, and their ratio were measured as indicators of vascular remodeling.

### 2.5 Immunohistochemistry

Immunohistochemical analysis was conducted as previously described (Ren et al., 2020). The thoracic aorta was prefixed, and the paraffin-embedded sections were washed with PBS, blocked with blocking buffer, and treated with primary antibodies for 24 h at 4°C, then primary antibodies were washed out, followed by incubation with the secondary antibodies for 30 min at room temperature. Positive cells were visualized using 3,3-diaminobenzidine. Then, images were obtained with an Olympus microscope.

### 2.6 Evaluation of Vascular Smooth Muscle Cells Proliferation

The proliferation ability of VSMCs was assessed by CCK-8 assay, 5-ethynyl-2ʹ-deoxyuridine (EdU) incorporation assay, and PCNA expression (Ren et al., 2017). Cells were seeded into 96-well plates (5 × 10^4^/ml) for 24 h. After 24 h of starvation in serum-free medium, the cells were then exposed to normal glucose, high glucose (25 mmol/L), or high glucose with various doses of Nar (50, 100, and 150 μmol/L) for 24 h (Wu et al., 2020). Appropriate amount of Nar was dissolved in DMSO, making it at the concentration of 1 mmol/L, which was then diluted with the corresponding DMEM into 50, 100 and 150 μmol/L. After cultivation, 10 μL of CCK-8 (APE × Bio) was added to each well. After incubation at 37°C in a 5% CO2 atmosphere for 2.5 h, absorbance was examined by a microplate reader (Thermo Fisher) at 450 nm. For the EdU incorporation assay, cells were seeded in six-well plates and cultured up to 80–95% density, followed by starvation and incubation with diverse DMEM or Nar for 24 h. EdU incorporation assay was performed in accordance with the manufacturer’s instructions. EdU-positive cells were examined by flow cytometry (FCM). Besides, PCNA, acting on chromatin as a platform for various proteins in the processes of DNA replication, was detected by western blot as a marker of cell proliferation.

### 2.7 Cell Migration Assay

For the wound scratch assay, VSMCs were seeded into six-well plates and cultured up to 80–90% confluence, the cells were then starved in starvation medium for 24 h. A single scratch wound was generated using a 1 mL pipette tip, after which the cells were subjected to different stimulations for an additional 12, 24, or 48 h. Cell migration was assessed by measuring scratch area with ImageJ software.

A modified transwell assay in 24-well plates was used to analyze VSMC migration. VSMCs (5 × 10^4^/ml) were resuspended in serum-free medium and 200 μL was added to the upper chamber. The lower chamber was filled with 500 μL of DMEM (10% FBS) with normal glucose, high glucose, and/or different concentrations of Nar. After 24 h of incubation, cells migrated to the lower face of the chamber. The number of migrated cells was clarified by violet staining, as described previously (Zhang et al., 2019).

### 2.8 Immunofluorescence Analysis

Immunofluorescence analysis was performed as previously described (Zang et al., 2019). Briefly, VSMCs were plated on glass coverslips in six-well plates, cultured up to 40–50% confluence, and treated with 4% paraformaldehyde for 20 min. They were then washed with PBS and permeabilized with 0.2% Triton X-100 for 5 min, blocked with 5% BSA at room temperature for 30 min, and incubated with the primary antibodies (1:100) overnight at 4°C. The cells were washed with PBS and incubated with FITC/TRITC-conjugated fluorescence secondary antibody (1:500) for 2 h at 37°C. The nuclei were stained with DAPI (1:1000) for 15 min at room temperature. Immunofluorescence images were acquired with an Olympus cellSens Entry (Olympus, Tokyo, Japan).

### 2.9 qRT-PCR

Total RNA of each group of VSMCs was extracted using the miRNA isolation Kit (Omega, United States) and quantified using the NanoDrop one Spectrophotometer (Thermo-Fisher, United States). cDNA was carried out using a cDNA synthesis Kit (Thermo-Fisher, United States) according to the instructions. Quantitative Reverse Transcriptase PCR (qRT-PCR) samples were prepared using SYBR Green PCR Kit (500), and amplified by using LightCycler96 Real-time fluorescent quantitative PCR Instrument (Roche, Switzerland). Relative mRNA expression was normalized to the control mRNA expression using the ΔΔCt method.

### 2.10 Western Blotting

Cellular protein was extracted from the diversely treated VSMCs with radioimmune precipitation assay (RIPA) lysis buffer, as previously reported (Jia et al., 2019). Protein concentration was measured using a bicinchoninic acid (BCA) protein assay kit. Equal amounts of the protein samples were separated by 10% or 12% SDS-PAGE and transferred to a PVDF membrane, followed by blocking with 5% BSA or dry milk in Tris-buffered saline (TBS) for 2 h at room temperature. The primary antibodies (1:1000 dilution) were incubated overnight at 4°C; after washing with TBST (TBS containing 0.1% Tween 20) three times for 10 min each, treatment with the secondary antibodies was applied (1:10,000 dilution; Zhongshan Jinqiao, China) for 2 h. Following three washes with TBST, the membranes were incubated with enhanced chemiluminescence (ECL) reagent and exposed to the Odyssey Infrared Imaging System. Images were analyzed by ImageJ software.

### 2.11 Collection of Nar’s Potential Targets

The molecular structure of Nar was confirmed using PubChem (https://pubchem.ncbi.nlm.nih.gov/), which was imported into the Swiss Target Prediction database (http://swisstargetprediction.ch/) and TargetNet database (http://targetnet.scbdd.com) for compound target prediction analysis (Ai et al., 2020).

### 2.12 Collection of Diabetic Angiopathy-Related Disease Target Genes

The term “diabetic angiopathies” was used as a keyword to acquire important target genes of diabetic angiopathies with the following databases: DisGeNET (https://www.disgenet.org/), GeneCards database (https://www.genecards.org/), OMIM database (https://www.omim.org/), and the Comparative Toxicogenomics Database (CTD, http://ctdbase.org/) (Xiao et al., 2021). Then, the UniProt database (https://www.uniprot.org/) was used to convert the target name into an official gene symbol by inputting the target name and limiting the species to human beings (Xiao et al., 2021). Finally, the database search results were integrated and the duplicates were deleted to acquire all target genes of diabetic angiopathies.

### 2.13 Common Target Acquisition and PPI Network Construction

The common targets of diabetic angiopathies and Nar were used in the OmicShare tools (https://www.omicshare.com/) to create a Venn map. They were then used in the String database (https://string-db.org/) to obtain the relevant information of protein–protein interactions (PPI), which was subsequently demonstrated and visualized by Cytoscape 3.7.0 software. Meanwhile, the degree values of proteins and the key proteins within the PPI network were acquired using Cytoscape 3.7.0 software.

### 2.14 Functional Enrichment Analysis

GO and KEGG pathway analyses of the common proteins were performed using DAVID database (https://david.ncifcrf.gov/) as Excel and bubble charts. The results were sorted according to *p* value and count value.

### 2.15 RNA Extraction and Analysis of Transcriptomic Sequencing

RNA samples were extracted from diversely treated VSMCs (*n* = 3) using Trizol reagent, as previously described (Sun et al., 2021). VSMCs were incubated in different DMEM for 24 h as follows: high glucose (HG group) and high glucose with Nar (100 μmol/L) (Nar group). Then, the RNA samples were analyzed by NovelBio (Shanghai, China).

### 2.16 PPI Network and Pathway Enrichment Analysis of Transcriptomic Sequencing

The differentially expressed genes (DEGs) were acquired from transcriptomic sequencing by comparing the Nar group with the HG group, which are potential targets of Nar in the treatment of diabetic angiopathies. To analyze the information on the interactions of the candidate DEGs, String database (https://string-db.org/) and Cytoscape 3.7.0 software were used. The KEGG pathways were analyzed according to the results of the transcriptomic sequencing.

### 2.17 Molecular Docking

Molecular docking was performed to analyze the interaction between Nar and core targets, which was conducted using LibDock. The complexes of the targets were downloaded from the Protein Data Bank (PDB) database according to PDBbind benchmark dataset (version 2020, www.pdbbind.org.cn) (Ren et al., 2018; Rezaei et al., 2022), and the cognate ligand docking and inhibitor ligand docking were performed as the positive control test. The 3D structures of Nar and inhibitors were downloaded from the PubChem database (https://pubchem.ncbi.nlm.nih.gov/). Docking programs were operated using LibDock with protein-molecules interactions, a higher LibDock score means a greater capacity for ligand-protein binding, as obtained in Discovery Studio 2016 (Li et al., 2021). The binding results were visualized as 3D and 2D diagrams using Discovery Studio Visualization version 4.5 (Accelrys, Inc., San Diego, CA, United States).

### 2.18 Statistical Analysis

Trials were directed in a double-blinded and randomized fashion. All data are presented as mean ± SD. Student’s paired *t*-test was used for comparisons between two groups. One-way ANOVA was used for multiple comparisons. *p* < 0.05 was considered statistically significant.

## 3 Results

### 3.1 Protective Effect of Nar Against Diabetic Angiopathies *in vivo*

Firstly, type 1 diabetic C57BL/6 mice model was used to assess the effect of Nar on diabetic vascular remodeling. After being injected with STZ for consecutive 5 days, 36 mice with blood glucose > 16.6 mmol/L were considered diabetic and randomly divided into three groups, DM group, DM + LN group and DM + HN group. According to the blood glucose and body weight monitoring, the blood glucose of DM group was significantly increased for continuous 12 weeks compared with Control group, but the blood glucose of Nar group including DM + LN and DM + HN group had no significant differences compared with DM group (Supplementary Figure 1A). At the same time, the body weight of DM group was significantly decreased compared with Control group, and the body weight of DM + LN and DM + HN were consistent with DM group (Supplementary Figure 1B).

**FIGURE 1:**
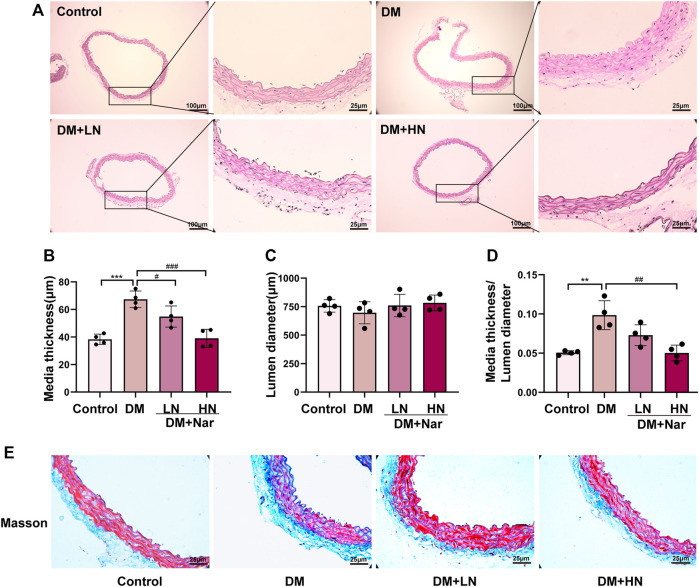
Protective effect of Nar against diabetic angiopathies *in vivo*. **(A)** HE staining of thoracic aorta from diverse groups of mice. **(B)** Bar graph of media thickness of aorta in diverse groups. **(C)** Bar graph of lumen diameter of aorta in diverse groups **(D)** Bar graph of HE staining analysis the ratio of media thickness/lumen diameter in aorta. ^**^ *p* < 0.01, ^***^ *p* < 0.001 compared with Control group, ^#^ *p* < 0.05, ^##^ *p* < 0.01, ^###^ *p* < 0.001, compared with HG group. **(E)** Representative images of Masson’s staining of the aorta (magnification: 400×).

According to HE staining, it revealed a higher media thickness of aorta in DM group, compared with Control group, and the media thickness of aorta in DM + LN and DM + HN group was significantly less than DM group (Figures 1A–D). At the same time, Masson’s trichrome staining showed a severer fibrosis in DM group, which was alleviated significantly after treatment with Nar (Figure 1E).

**FIGURE 2:**
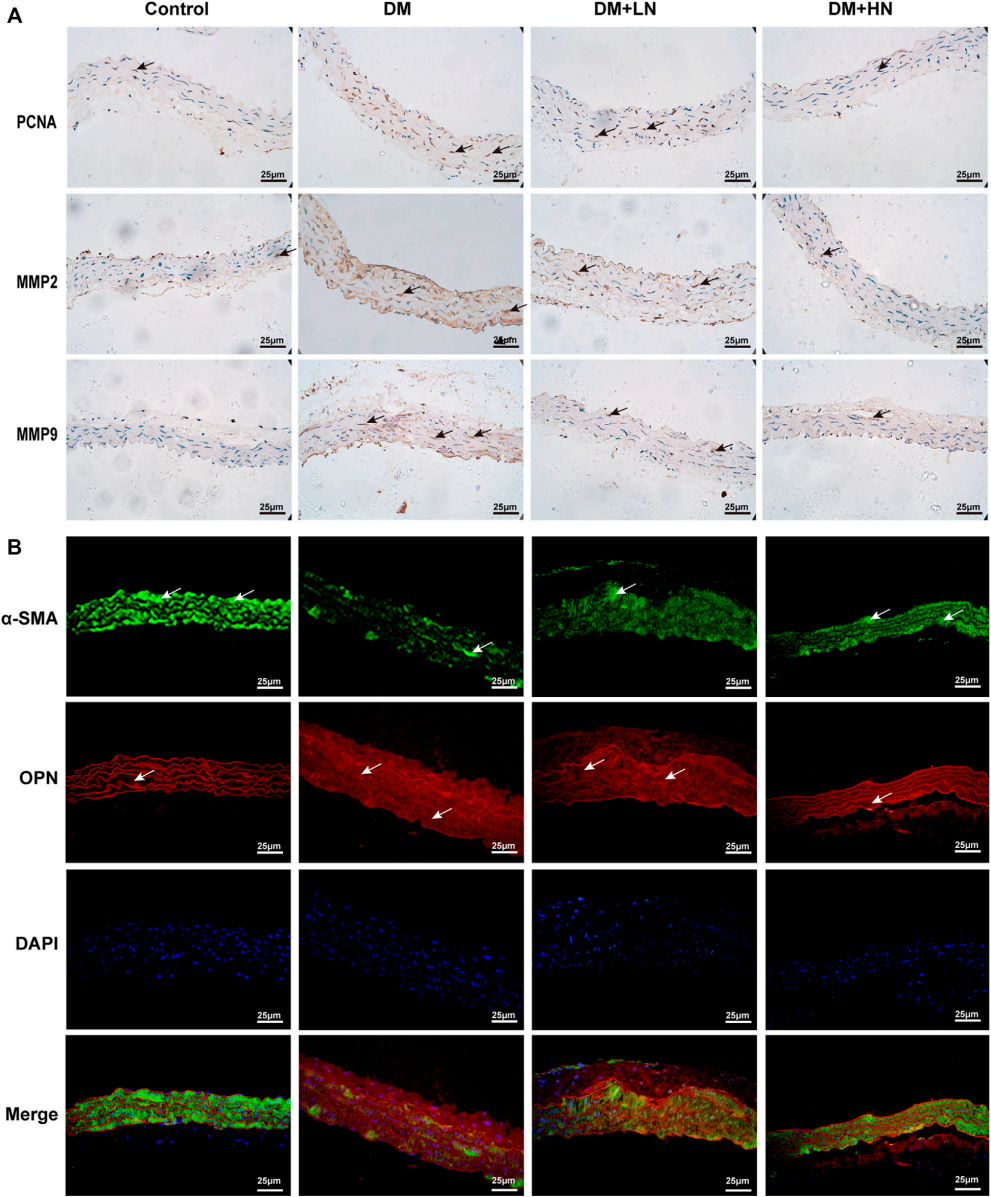
**(A)** Nar inhibited diabetes induced VSMCs’ proliferation, migration and phenotype switching *in vivo*. Immunohistochemical analysis of PCNA, MMP2, and MMP9 expression in the aorta in diverse groups (magnification: 400×). **(B)** Immunofluorescence staining of α-SMA and OPN expression in diverse groups of mice.

Whether Nar has the effect of anti-proliferation and anti-migration on VSMCs, immunochemistry staining was exerted to detect the expression of related proteins, PCNA, MMP2, and MMP9. According to the result of immunochemistry staining, the expression of PCNA, MMP2, and MMP9 were increased in DM group, and treatment with Nar (DM + LN, DM + HN) decreased the expression of PCNA, MMP2, and MMP9 (Figure 2A).

Moreover, to confirm the effect of Nar reversed VSMCs dedifferentiation, immunofluorescence staining was used to detected the expression of α-SMA, a marker of characteristic contractile phenotype of VSMC, and OPN, a marker of synthetic phenotype of VSMCs. As shown in Figure 2B, there was a lower expression of α-SMA in DM group compared with Control group, but a higher expression in DM + LN and DM + HN groups compared with DM group, on the contrary, the expression of OPN was highly increased in DM group, and reduced significantly in DM + LN and DM + HN groups.

### 3.2 Nar Inhibited High-Glucose-Induced Vascular Smooth Muscle Cells Proliferation

**FIGURE 3:**
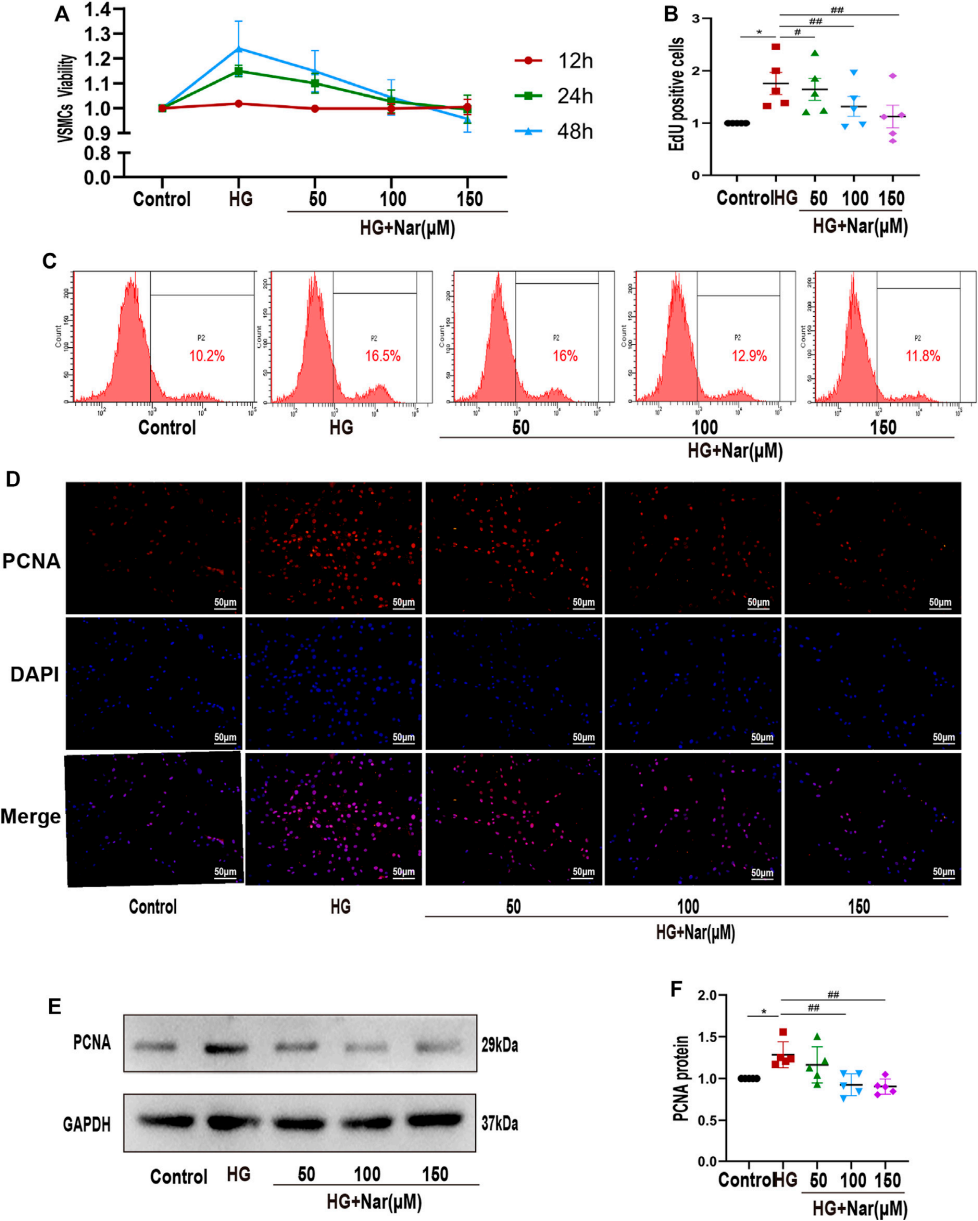
Nar inhibited high-glucose-induced VSMC proliferation. **(A)** CCK-8 assay showed that Nar reduced the viability of high-glucose-induced VSMCs at 24 and 48 h, *p* < 0.05. **(B,C)** Flow cytometry showed a lower cell proliferation rate in the Nar group than in the HG group. **(D)** Immunofluorescent staining of PCNA (magnification: 200×). **(E,F)** Western blot analysis showed PCNA expression associated with different treatments of VSMCs, ^*^ *p* < 0.05 compared with Control group, ^##^ *p* < 0.01 compared with HG group.

Firstly, VSMC was isolated and characterized as shown in Supplementary Figure 2A,B, and only passages 3 to passages 6 were used in cell experiments. To evaluate the influence of Nar on VSMCs under high-glucose conditions, we first confirmed the cytotoxic effects of Nar on VSMCs at 200 μmol/L, but not at other concentrations (50, 100, and 150 μmol/L) (Supplementary Figure 2C). Then, VSMCs were stimulated with a high level of glucose along with different concentrations of Nar (50, 100, and 150 μmol/L) for 24 h to examine cell proliferation by CCK-8 assay and flow cytometry. CCK-8 assay showed that high glucose can lead to higher viability of VSMCs, while Nar decreased the high glucose-induced cell viability (Figure 3A). Flow cytometry indicated that Nar at concentrations of 50, 100, and 150 μmol/L inhibited the high-glucose-induced proliferation of VSMCs (Figures 3B,C). At the same time, incubation with a high level of glucose induced the upregulation of PCNA, considered a marker of cellular proliferation, and Nar suppressed the upregulation of PCNA as reflected by the fluorescence intensity (Figure 3D) and protein expression (Figures 3E,F) in high glucose-incubated VSMCs.

### 3.3 Nar Inhibited High-Glucose-Induced Vascular Smooth Muscle Cells Migration

**FIGURE 4:**
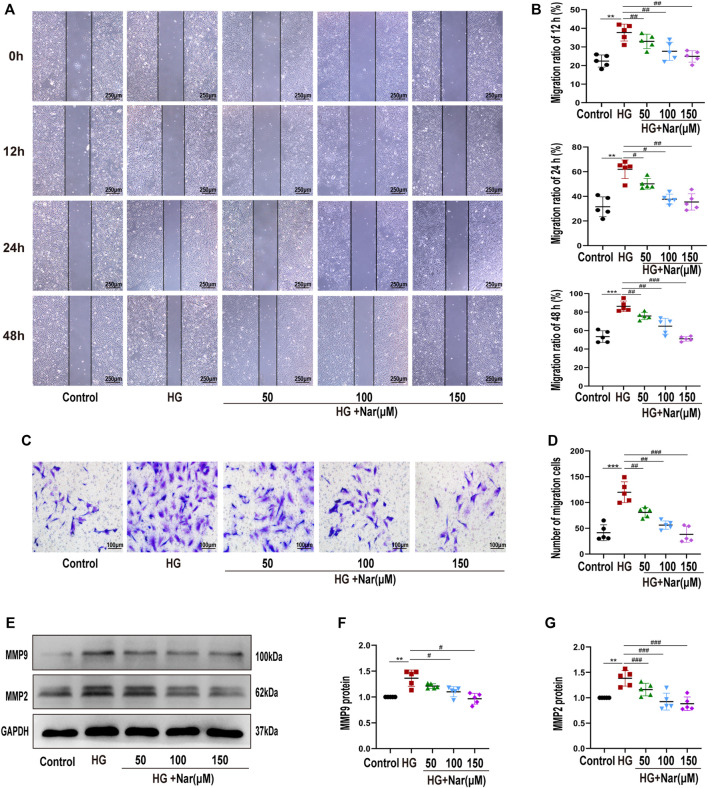
Nar inhibited high-glucose-induced VSMC migration. **(A,B)** Wound healing assay of VSMC migration at 12, 24, and 48 h (magnification: 40×). **(C,D)** Transwell migration assay of Control group, HG group, and groups with diverse doses of Nar (magnification: 200×). **(E–G)** Western blot analysis of MMP2 and MMP9 expression. ^**^ *p* < 0.01 compared with Control group, ^##^ *p* < 0.01 compared with HG group.

Next, to explore whether Nar abrogated VSMCs migration in response to high glucose, wound healing assay and transwell assay were exerted. As shown in Figures 4A,D, incubation of VSMCs with high glucose resulted in an increase of their scratch area, however, Nar counteracted the high-glucose-induced migration of VSMCs, as evidenced by wound healing assay (Figures 4A,B) and Transwell migration assay (Figures 4C,D). Markers such as MMP9 and MMP2 are considered to be involved in cell migration (Shen et al., 2021). Consistent with this, treatment with Nar mitigated the upregulation of MMP9 and MMP2 protein levels in high-glucose-incubated VSMCs (Figures 4E–4G).

### 3.4 Nar Abrogated High-Glucose-Induced Vascular Smooth Muscle Cells Dedifferentiation

**FIGURE 5:**
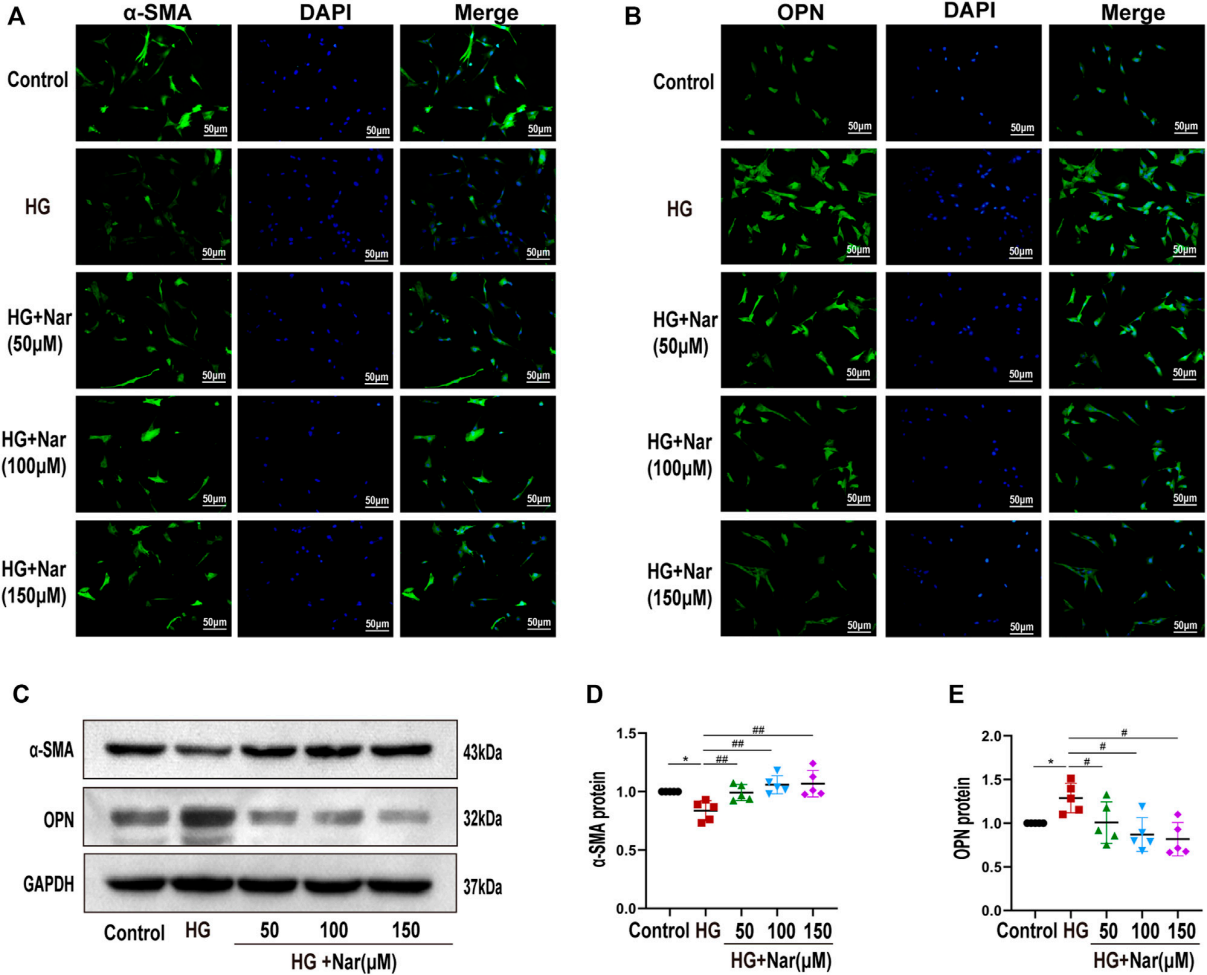
Nar abrogated high-glucose-induced VSMC dedifferentiation. **(A,B)** Immunofluorescent staining of α-SMA and OPN (magnification: 200×). **(C–E)** Western blot analysis of α-SMA and OPN expression. ^*^ *p* < 0.05 compared with Control group, ^#^ *p* < 0.05, ^##^ *p* < 0.01 compared with HG group.

VSMCs dedifferentiation plays a vital role in the proliferation and migration of VSMCs as well as vascular dysfunction. To detect whether Nar affected high-glucose-induced VSMC dedifferentiation, we examined the expression of α-SMA and OPN of VSMCs in terms of fluorescence intensity and protein levels. Our results indicated that high glucose can induce the change of VSMC phenotype from differentiated to dedifferentiated cells, as evidenced by the increased expression of OPN, a marker of a synthetic phenotype, along with decreased expression of α-SMA, a marker of a contractile phenotype. As shown in Figures 5A,C,D, the expression of α-SMA was significantly reduced, and after treating with different concentrations of Nar, the expression of α-SMA was gradually increased. Simultaneously, as shown in Figures 5B,C,E, the expression of OPN was significantly increased, while decreased after incubation with diverse concentrations of Nar. Our results demonstrated that incubation with Nar significantly antagonized the VSMCs dedifferentiation response to high glucose.

### 3.5 Results of Network Pharmacology Analysis

#### 3.5.1 Collection of Nar Targets and Diabetic Angiopathy Targets

**FIGURE 6:**
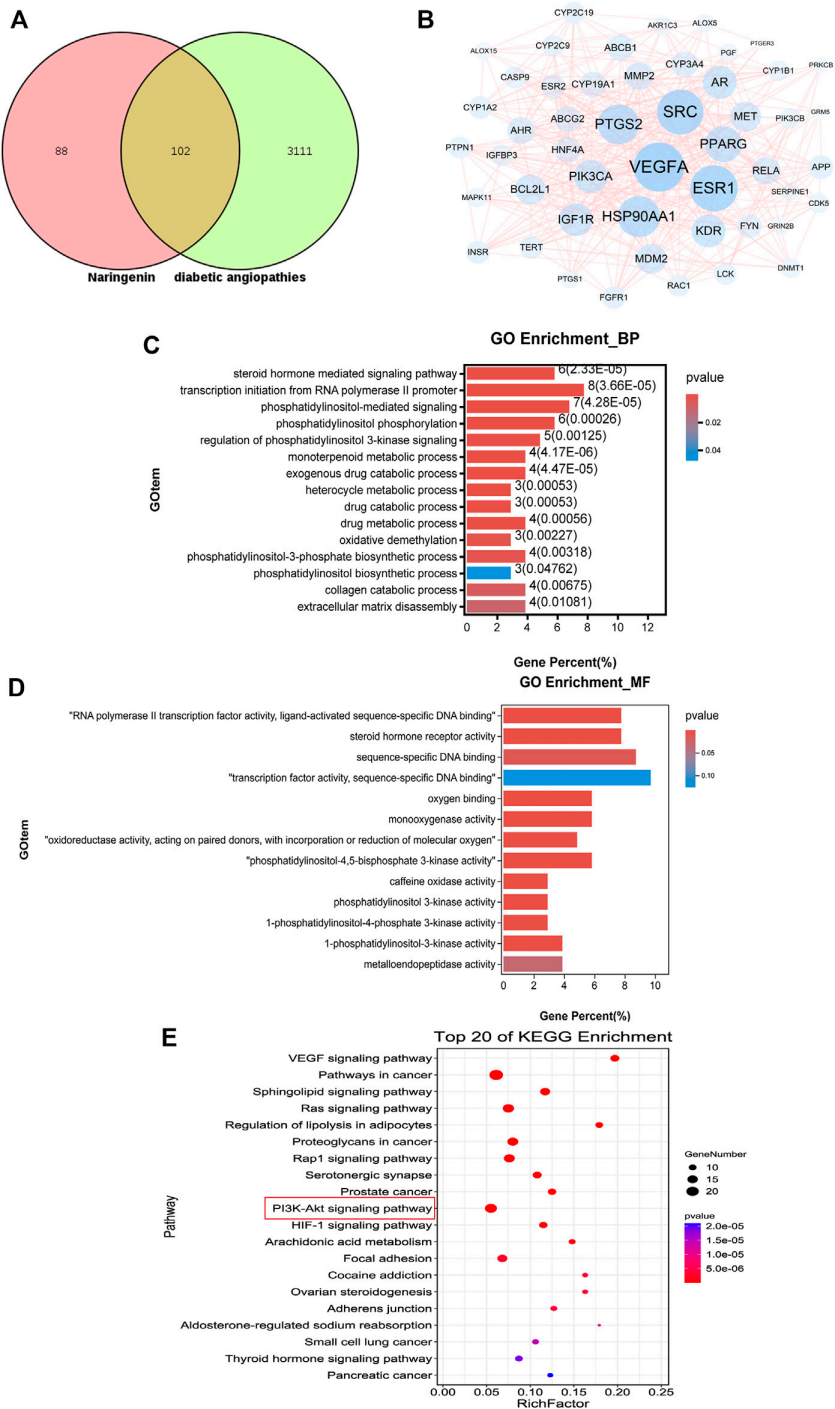
Results of network pharmacology analysis. **(A)** Venn diagram shows the number of common targets between Nar and diabetic angiopathies. **(B)** Protein–protein interaction (PPI) network of the common targets according to degree value analyzed by Cytoscape software. **(C)** GO enrichment (BP) analysis of network pharmacology. **(D)** GO enrichment (MF) analysis of network pharmacology. **(E)** Top 20 most enriched KEGG categories for the common targets shows the vital Nar-related signaling pathway against diabetic angiopathies according to network pharmacology analysis.

According to the Swiss target prediction database and TargetNet database, 190 Nar-related targets were collected. A total of 3213 diabetic angiopathy-related genes were acquired from the GeneCards, OMIM, DisGeNet, and CTD databases after removing duplicate genes, and 102 common targets were obtained, as shown in Figure 6A. The PPI network of these 102 potential targets was constructed via the String database, according to the degree, top 50 of them were visualized using Cytoscape 3.7.0 as shown in Figure 6B. The top 10 proteins were as follows, VEGFA (degree 51), PTGS2 (degree 47), Src (degree 45), ESR1 (degree 41), HSP90AA1 (degree 37), AR (degree 34), APP (degree 33), KDR (degree 30), PIK3CA (degree 29) and MMP2 (degree 27).

### 3.5.2 GO and KEGG Pathway Analyses

To identify the possible mechanism by which Nar suppresses diabetic angiopathies, 102 predicted targets were subjected to GO and KEGG enrichment analyses. GO enrichment analysis including biological process (BP) and molecular function (MF) analysis identified the involvement of the targets in the response to phosphatidylinositol-3-phosphate biosynthetic process, extracellular matrix disassembly, and phosphatidylinositol 3-kinase activity, among others, which are closely related to cell proliferation and migration (Figures 6C,D). According to the *p*-values (*p* < 0.05) and count values, the top 20 KEGG pathways involved in the alleviation of diabetic angiopathies by Nar were identified as major pathways (Figure 6E) intimately associated with proliferation and migration, including the VEGF signaling pathway and PI3K-Akt signaling pathway.

### 3.6 Results of Transcriptomic Sequencing and Data Analysis

#### 3.6.1 DEGs Reversed by Nar

**FIGURE 7:**
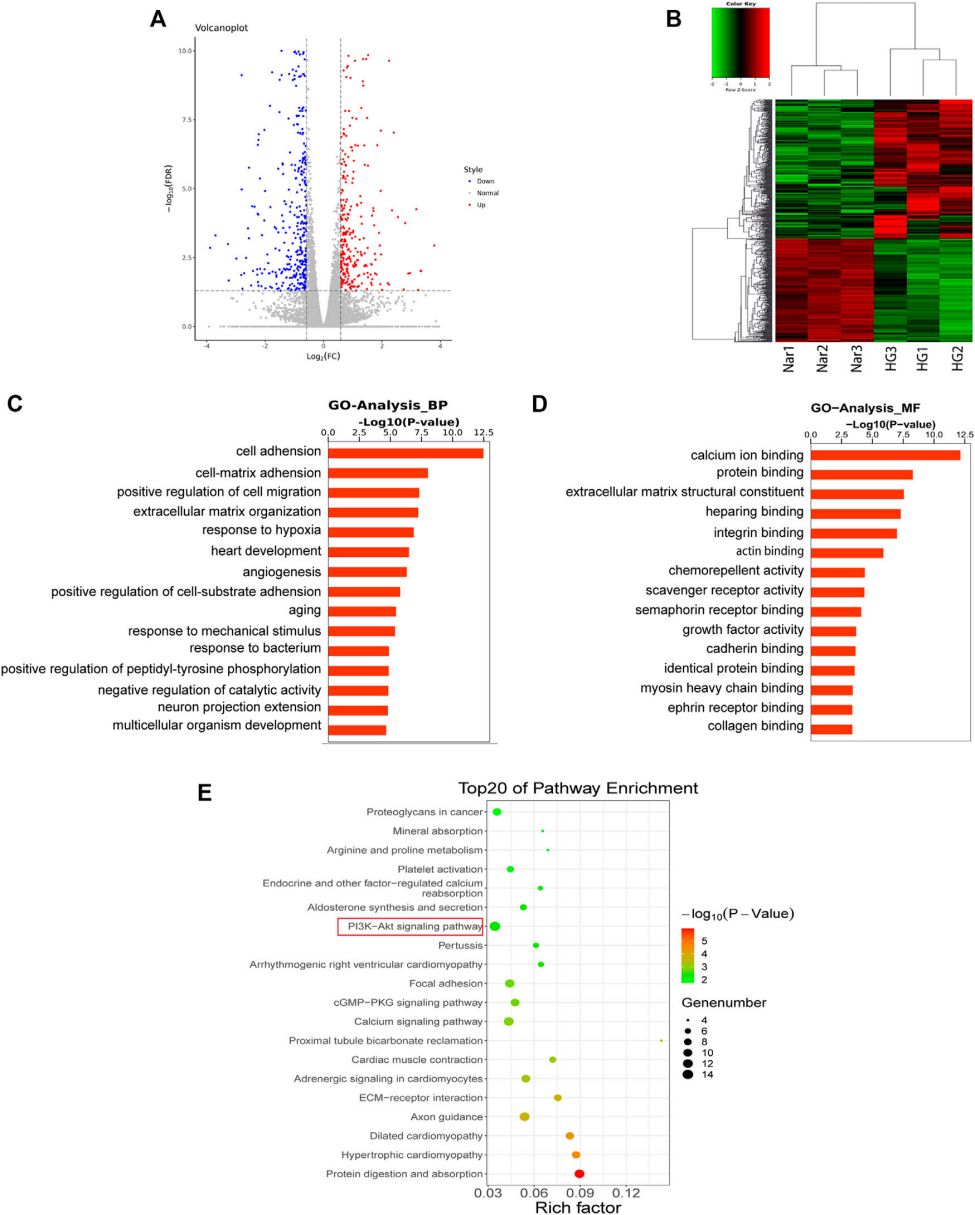
Results of transcriptomic sequencing and data analysis **(A)** A volcano plot indicates the mRNA expression profile of the Nar group vs the HG group. **(B)** Heatmap shows the clustering of mRNAs between the Nar and HG groups of VSMCs. Up- and downregulated genes are colored in red and blue, respectively. **(C)** GO enrichment (BP) analysis of transcriptomic analysis. **(D)** GO enrichment (MF) analysis of transcriptomic analysis. **(E)** Top 20 most enriched KEGG categories for the downregulated genes shows the vital Nar-related signaling pathway against diabetic angiopathies according to transcriptomic analysis.

To further explore the mechanism by which Nar acts against diabetic angiopathies, we conducted transcriptomic sequencing on VSMC samples incubated with high glucose (HG) or high glucose with Nar (Nar). A total of 711 DEGs showed significant change of expression due to Nar compared with the level with HG (*p* < 0.05, |Log2FC| > 0.585), 303 of which were upregulated and 408 were downregulated in the Nar group (Figures 7A,B).

### 3.6.2 GO and KEGG Pathway Analyses

GO and KEGG enrichment analyses of the above-mentioned up- and downregulated DEGs were conducted. The GO enrichment of downregulated genes was shown in Figures 7C,D, which identified cell–matrix adhesion, positive regulation of migration, and angiogenesis, factors that are intimately related to cell proliferation and migration.

The results of KEGG enrichment analysis of 408 downregulated DEGs were listed in Figure 7E, which identified the cGMP/PKG signaling pathway and PI3K/Akt signaling pathway were closely related to cell proliferation and migration. Interestingly, we found that the same KEGG pathway, namely, the PI3K/Akt pathway, was identified both in network pharmacology and transcriptomic analyses, indicating its potentially vital role in Nar’s effects against VSMC proliferation and migration induced by high glucose. Thus, the PI3K/Akt signaling pathway was regarded as the key pathway of Nar protected against diabetic angiopathies.

The genes enriched in the PI3K/Akt pathway were listed in Supplementary Table 1, including ITGA4, TNC, IGF1, and CREB5 et al., among them, CREB5, which was found in the downstream part of this pathway and was downregulated by Nar. At the same time, VEGFA, Src and KDR, located in the upstream part of the PI3K/Akt pathway, were identified as key targets included among the top 10 targets from the network pharmacology analysis.

### 3.7 Results of Molecular Docking Analysis

Molecular docking was carried out to investigate the potential binding modes and interactions between Nar and its key targets VEGFA (PDB: 1VPP), Src (PDB: 5XP7), and KDR (PDB: 3VHK). As shown in Table 1, the LibDock score between VEGFA and Nar was 98.7813, and the LibDock score between VEGFA and inhibitors (PTC299 and Erdafitinib) were 99.4048 and 117.326. As to Src, it had LibDock score of 104.866 interacted with Nar, 128.602 interacted with its cognate ligand (8C6), and 111.639 interacted with the specific inhibitor (Src inhibitor 1). And the interaction between KDR and Nar was evaluated based on the dock score of 111.412 interacted with Nar, 120.848 interacted with cognate ligand (BPK), and 121.494 interacted with specific inhibitor (Linifanib). So, comparing the LibDock Score of VEGFA Src, and KDR bound with different molecules, the LibDock Score of Src and KDR bound with Nar had no significant differences with those of Src and KDR bound with their cognate ligands and inhibitors, which demonstrated that Nar had good binding affinity with Src and KDR. At the same time, the LibDock Score of VEGFA bound with Nar had no significant differences with those of VEGFA bound with its specific inhibitors, which illustrated that Nar had good binding affinity with VEGFA (Table 1).

**TABLE 1:** LibDock analysis of core targets bound with different molecules.

Target	PDB	LibDock score		
		Naringenin	Cognate ligand	Inhibitor
VEGFA	1VPP	98.7813	99.4048	117.326
Src	5XP7	104.866	128.602	111.639
KDR	3VHK	111.412	120.848	121.494

**FIGURE 8:**
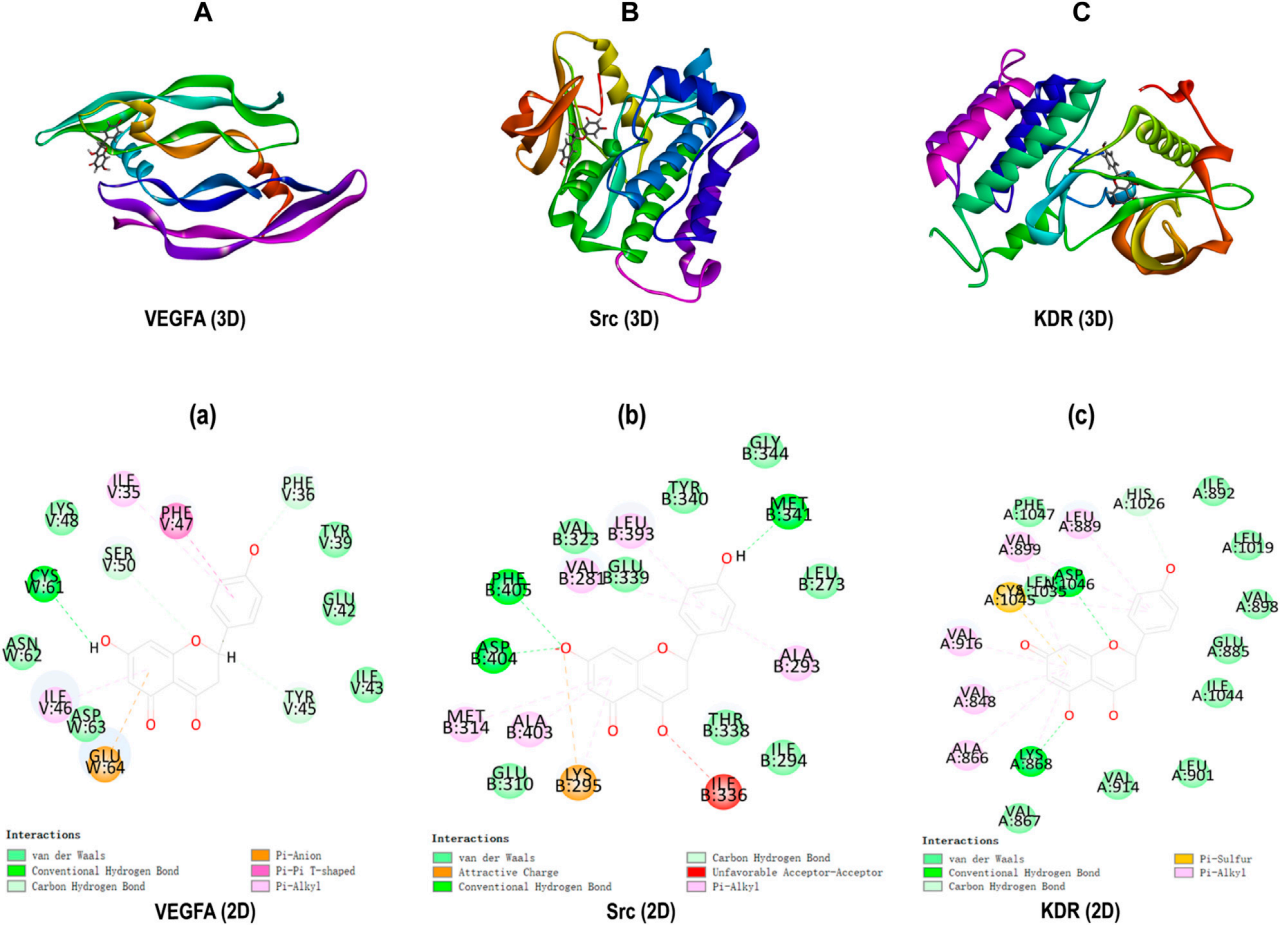
Molecular docking. **(A-C)** 3D docking for Nar in the active binding pocket of VEGFA, Src and KDR. **(a-c)** 2D interaction patterns between Nar and VEGFA, Src and KDR.

The interactions among proteins and molecules were shown in Figure 8, according to Figures 8A,a, the interactions between Nar and VEGFA included the formation of π-anion interaction with GLU64 residue, π-π stacked interaction with PHE47 residue, π-Alkyl interaction with ILE35 and ILE46 residues, and conventional hydrogen bond form with its ASP63 residue. In addition, as shown in Figures 8B,b, the attractive charge interaction with LYS295 residue, unfavorable acceptor-acceptor interaction with ILE336 residue, π-alkyl interaction with LEU393, VAL281, MET314, et al. residues, and hydrogen bond with PHE405, ASP404 and MET341 residues were involved in the interactions between Nar and Src. Besides, Figures 8C showed Nar could interact with KDR through π-sulfur interaction with CYS1045 residue, hydrogen bond with ASP1046 and LYS868 residues, and π-alkyl interaction with LEU889, VLA899, ALA866, et al. residues.

### 3.8 Regulatory Effect of Nar on PI3K/Akt/CREB5 Signaling Pathway

**FIGURE 9:**
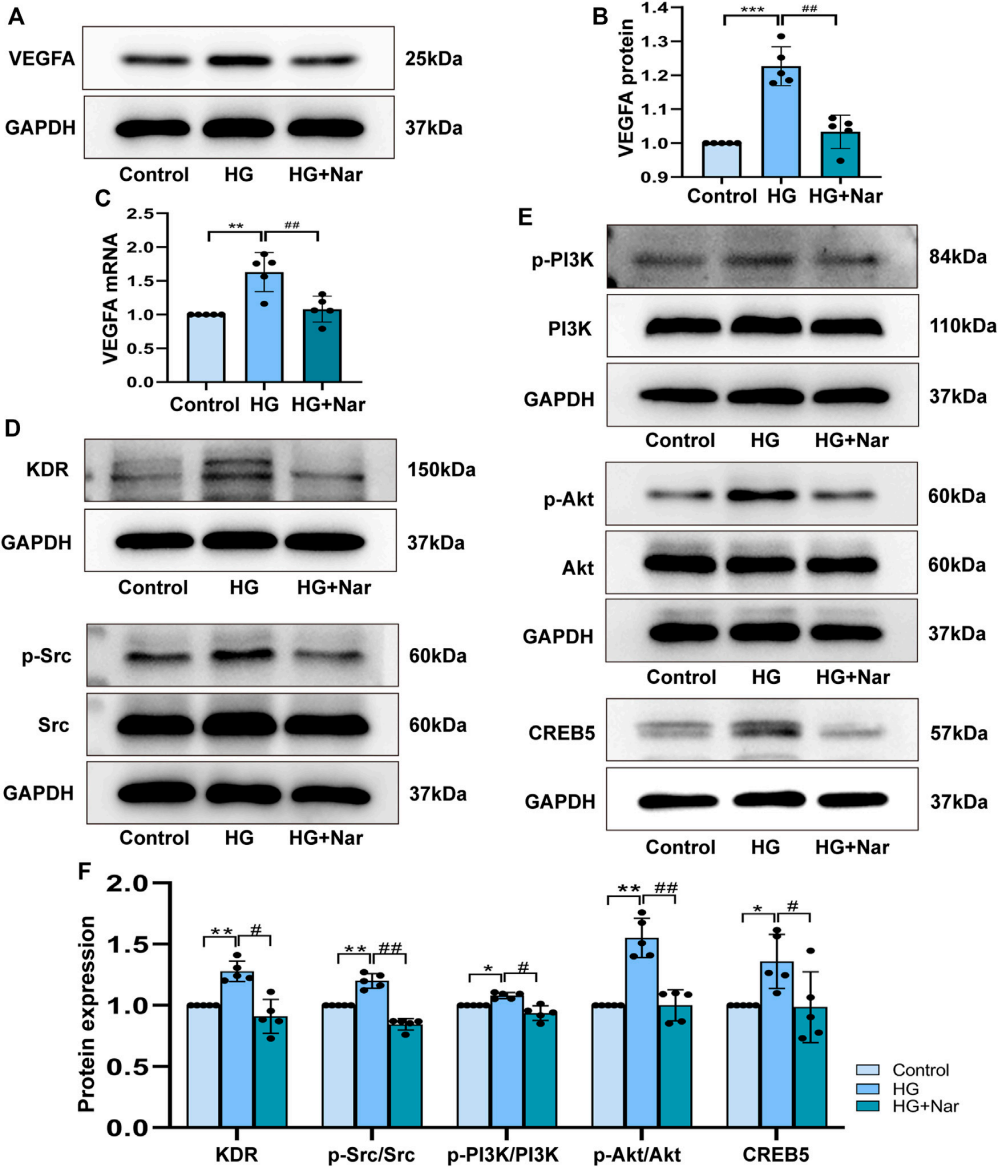
Nar down-regulated the expression of core target VEGFA and PI3K/Akt/CREB5 signaling pathway. **(A)** Western blot detected the protein expression of VEGFA. **(B)** Analysis of VEGFA protein expression. **(C)** Analysis of VEGFA mRNA expression. **(D,E)** Nar suppressed KDR, p-Src, p-PI3K, p-Akt and CREB5 expression which upregulated by HG group. **(F)** Bar graph showed related protein expression analysis. ^*^ *p* < 0.05, ^**^ *p* < 0.01 compared with Control group, ^#^ *p* < 0.05, ^##^ *p* < 0.01 compared with HG group.

To identify whether Nar could inhibit VEGFA directly, qRT-PCR was used to detected mRNA expression of VEGFA, the primer sequences as follows, VEGFA: forward 5′-CCC​TGG​CTT​TAC​TGC​TGT​ACC-3′; reverse 5′-CTT​CAT​GGG​CTT​TCT​GCT​CCC-3′. GAPDH: forward 5′-GAC​ATG​CCG​CCT​GGA​GAA​AC-3′; reverse 5′-AGC​CCA​GGA​TGC​CCT​TTA​GT-3′. At the same time, VEGFA protein expression was detected. As shown in Figures 9A–C, high glucose could induce high expression of VEGFA, and Nar could downregulate the mRNA and protein expression of VEGFA. To confirm that the anti-proliferation and anti-migration effects of Nar were related to PI3K/Akt/CREB5 signaling pathway, the expression of related proteins, including KDR, Src, p-Src, PI3K, p-PI3K, Akt, p-Akt, and CREB5 was detected. The results showed that the expression of KDR, p-Src, p-PI3K, p-Akt, and CREB5 was significantly upregulated by high glucose, and downregulated by Nar in the presence of high glucose (Figures 9D–F).

## 4 Discussion

In diabetic patients, the excessive proliferation of VSMCs induced by abnormally elevated blood glucose can cause and exacerbate macrovascular and microvascular diseases, such as hypertension, atherosclerosis, and restenosis (Kay et al., 2016). Acute increases of reactive oxygen species (ROS) and inflammatory cytokines caused by chronic hyperglycemia in diabetic patients have devastating effects on vascular function (Chapple et al., 2013). Additionally, the gradual accumulation of superfluous metabolites (free fatty acids, glycation end products, O-GlcNAcylation, etc.) may also promote the extreme differentiation, proliferation, and migration of VSMCs (Byon and Kim, 2020; Shi et al., 2020). The prevention and treatment of diabetic vascular complications mainly includes the following aspects: self-management and drug therapy, including antihyperglycemic, lipid-regulating, antihypertensive and anti-platelet drugs. In addition, our findings may provide new consideration for the molecular mechanism underlying the action of naringenin.

Nar, a compound with various pharmacological activities, like anti-inflammatory, anti-tumor, anti-diabetic and anti-proliferation effects. Some small-scale clinical studies have showed that the naringenin with a dosage ranging between 600 and 800 μmol/L/day has the ability to improve endothelial function in different patient groups. For example, Rendeiro et al., reported that the flow-mediated dilatation of brachial artery was improved after long-term daily supplementation with orange flavanone beverages in clinic study (Rendeiro et al., 2016). Habauzit et al. reported that endothelial dysfunction was improved after supplementation with long-term daily grapefruit juice/day containing about 480 μmol/L naringenin glycoside (Habauzit et al., 2015). Besides, Rebello et al., found that the single doses of 150, 300, 600, and 900 mg of naringenin was safe and tolerant in small-scale healthy adults (Rebello et al., 2020). These previous studies have provided valid basis for the clinical application of Nar.

α-Smooth muscle actin (α-SMA), as a particularly contractile protein, is a specific gene marker of smooth muscle cells (SMCs) (Goumans and Ten Dijke, 2018). Osteopontin (OPN) is involved not only in the metastatic phenotype but also in the synthetic phenotype of VSMCs, and can be highly activated under particular physiological and pathological conditions such as inflammation and imbalanced immunity (Denhardt et al., 2003; Zhang M. et al., 2020). Under physiological conditions, VSMCs preferentially adopt the differentiated and contractile phenotype, rather than the dedifferentiated and synthetic phenotype. Conversely, under pathological conditions, VSMCs can transform into the synthetic phenotype characterized by the high expression of OPN and low expression of α-SMA (Lin et al., 2018). In our study, we first found that Nar clearly decreased the media thickness and fibrosis of the aortic arch and modified the promiscuous and disordered smooth muscle cells in a type 1 diabetic (T1D) mouse model (Figures 1A–E).

Substantial evidence has shown that the transformation of VSMCs to a phenotype involving high synthesis contributes to increased proliferation, migration, and excessive production of extracellular matrix. Therefore, we continued to examine the effect of Nar on the proliferation and migration of VSMCs induced by high glucose. Proliferating cell nuclear antigen (PCNA), as a marker of VSMC proliferation, is an important factor in regulating DNA replication, DNA repair, sister-chromatid cohesion, and cell cycle control prominent inflammatory mediators, are a family of zinc-containing proteolytic enzymes that are involved in various processes, including growth, migration, angiogenesis, and metastasis of cancer cells (Strzalka and Ziemienowicz, 2011; Pittayapruek et al., 2016). MMP2 and MMP9 belong to gelatinases of MMPs’ main subgroups. MMP2 has been considered to be a potential mediator of VSMC migration, which can facilitate such migration by inducing a switch from a contractile to a synthetic phenotype (Belo et al., 2015). MMP9 is not only a biomarker of cancer cell invasion and tumor metastasis and expressed in various cancer cells, but also participates in the development of cardiovascular disease, lung disease, and diabetes (Mondal et al., 2020). In this study, immunohistochemical analysis showed that Nar significantly reduced the expression of PCNA, MMP2, and MMP9 proteins in the T1D model (Figure 2A). Simultaneously, we found that Nar not only significantly inhibited the proliferation by reducing the expression of PCNA and the number of Edu-positive cells (Figure 3), but also restrained the migration by decreasing the expression of MMP2 and MMP9 in the model of proliferating and migrating VSMCs induced by high glucose (Figure 4). Additionally, Nar significantly altered the dedifferentiation by upregulating α-SMA expression and downregulating OPN expression both *in vivo* (T1D model) (Figure 2B) and *in vitro* (high-glucose-induced VSMCs) (Figure 5) experiments. These results indicated that Nar not only significantly inhibited cell proliferation and migration but also modified the phenotype from a dedifferentiated and synthetic phenotype to a differentiated and contractile one both *in vivo* and *in vitro*.

To further explore the potential molecular mechanism behind the anti-proliferation and anti-migration effects of Nar on VSMCs, a transcriptomic approach was initially used to predict the potential signaling pathway. For transcriptomic sequencing analysis, 303 upregulated genes and 408 downregulated genes were found to be involved in the protective effect of Nar on VSMCs. The GO enrichment results for the differentially expressed genes (DEGs) showed particular associations with the biological processes of positive regulation of cell migration, extracellular matrix organization, and angiogenesis (Figures 7C,D). Meanwhile, the KEGG results of transcriptomic sequencing analysis showed that the PI3K/Akt signaling pathway et al. were involved in the protective effect of Nar on VSMCs (Figure 7E). Network pharmacology is another bioinformatic approach that has been considered as a novel research method for identifying putative targets and pharmacological mechanisms (Wei et al., 2016). Therefore, we also explored the potential targets and signaling pathways by using a network pharmacology-based method. Notably, GO enrichment analysis showed that the 102 overlaps between Nar and diabetic angiopathies were particularly related to the regulation of phosphatidylinositol 3-kinase signaling, including phosphatidylinositol-3-phosphate biosynthetic process and phosphatidylinositol 3-kinase activity (Figures 6C,D). Furthermore, KEGG pathway enrichment analysis provided the same result that the PI3K/Akt signaling pathway participated in the effects of Nar against diabetic angiopathies as in the network pharmacology and transcriptomic sequencing analyses (Figures 6E, 7E). Substantial evidence has indicated that the PI3K/Akt signaling pathway, MAPK signaling pathway, cytokine-cytokine receptor interaction, and cGMP/PKG signaling pathway are related to vasculopathy (Wang et al., 2013; Karar and Maity, 2011; Loppnow et al., 2011; Zhou et al., 2021). These reports are consistent with our results. Additionally, as shown in Figure 6A, there were 102 targets overlapping between drug targets (Nar) and disease targets (diabetic angiopathies) in the Venn diagram (Figure 6A), with VEGFA (degree 51), Src (degree 45), and KDR (degree 30) being the core targets according to the degree value in the PPI network (Figure 6B). Vascular endothelial growth factor A (VEGFA) is closely related to the proliferation and migration of endothelial cells by participating in angiogenesis by binding to KDR (VEGFR2) (Claesson-Welsh and Welsh, 2013). Src, as a downstream target of VEGFR, plays an important role in the regulation of angiogenesis by binding its SH2 domain to a tyrosine autophosphorylation site on EGFR (Park et al., 2007; Schenone et al., 2007). The interaction of VEGFA and VEGFR2 has been shown to contribute to abnormal angiogenesis by regulating various pathways, including proliferation through the PLC-γ/PKC/RAF/MEK/ERK signaling pathway, vascular permeability and survival via the PI3K/Akt signaling pathway, and migration through the p38-MAPK signaling pathway (Zhang A. et al., 2020). Considering the consistent results of transcriptomic analysis, network pharmacology analysis, and the scientific evidence obtained to date, we speculated that the core targets of VEGFA, Src and KDR, and the PI3K/Akt signaling pathway might be involved in the anti-proliferation and anti-migration effects of Nar.

For the core targets of VEGFA, Src and KDR, we initially used molecular docking to evaluate the binding affinity, and positive control test was performed as the benchmark. As shown in Figure 8 and Table 1, according to the LibDock score of proteins interacted with different ligands, Nar had good binding affinity with VEGFA, Src and KDR. Then, we continued to analyze the expression of VEGFA, Src and KDR, the results showed that Nar prominently reduced the mRNA and protein expression of VEGFA (Figures 9A–C), indicating VEGFA was inhibited by Nar directly. According to Figures 9D,F, the protein expression of Src and KDR was significantly downregulated by Nar, which consistent with the results of molecular docking. VEGFA, an important subunit of the VEGF family in vascular, was found in the upstream of PI3K/Akt pathway. The synthesis and secretion of VEGFA is greatly associated with the progression of many angiogenesis diseases (Doronzo et al., 2012). According to previous studies, VEGFA was high expressed in high glucose induced VSMCs, and the inhibition of VEGFA abrogated excessive VSMCs proliferation and migration (Natarajan et al., 1997; Chen et al., 2014). Besides, Chan KC, et al., and Wen X, et al. proved that PI3K/Akt could be activated by high glucose, resulting in a promotive cell proliferation and migration (Chan et al., 2012; Wen et al., 2022). These studies indicated that VEGFA mediated PI3K/Akt pathway could take part in the high glucose induced endothelial dysfunction.

It is reported that the overexpression of cAMP-responsive element binding protein 5 (CREB5) promotes the proliferation and migration of different cancer cells. Specifically, CREB5 has been shown to promote cell proliferation and correlate with a poor prognosis in hepatocellular carcinoma, while the lncRNA SNHG5 affects the cell proliferation, metastasis, and migration of colorectal cancer through regulating miR-132–3p/CREB5 (Wu et al., 2018). According to the DEGs identified in the transcriptomic analysis, we found that CREB5 was particularly associated with the PI3K/Akt pathway and significantly upregulated in the VSMCs induced by high glucose. However, the high level of CREB5 in the model of proliferating and migrating VSMCs induced by high glucose was clearly downregulated by Nar. Consistent with the results of transcriptomic analysis, the protein level of CREB5 was significantly reduced by Nar (Figures 9E,F). To the best of our knowledge, we are the first to discover that CREB5 is involved in the proliferation and migration of VSMCs induced by high glucose. Additionally, CREB5 has a close relationship with the PI3K/Akt pathway. Huang et al. reported that this pathway was upstream of CREB5, which participated in the proliferation, migration, and invasion of breast cancer (Huang et al., 2019). Hiroya et al. found that human coronary artery smooth muscle cells (CASMCs) preferentially proliferate rather than undergo apoptosis in a manner dependent on the downregulated expression of the bcl-2 gene family (bcl-xl and bfl-1/A1) through the PI3K and MAPK pathways in high-glucose-stimulated human CASMCs (Sakuma et al., 2002). VSMCs could undergo phenotypic transformation and increase their ability to migrate and proliferate through upregulation of the ERK1/2 and PI3K/Akt signaling pathways stimulated by high glucose (Shi et al., 2015). In a similar study, Fan et al. showed that the injection of hong jing tian could improve proliferation and migration by inhibiting the Akt pathway in high-glucose-induced vascular smooth muscle cells (Fan et al., 2019). In addition, Zhou et al. found that arctiin could attenuate the proliferation and arrest the cell cycle in the G0/G1 phase by inactivating VEGF and the PI3K/Akt signaling pathway in high-glucose-induced human retinal capillary endothelial cells (Zhou et al., 2020). These studies are consistent with our results. The experiments in the current study demonstrated that Nar could ameliorate the proliferation and migration by decreasing the expression of VEGFA proteins, and downregulating the downstream PI3K/Akt and CREB5 pathway.

## 5 Conclusion

In summary, in this study transcriptomic sequencing, network pharmacology analysis, and in the vitro and vivo experimental validation were used to illustrate the potential mechanism behind the anti-proliferation and anti-migration effects of Nar in a model of high-glucose-stimulated VSMCs. The obtained results showed that Nar can significantly attenuate the proliferation and migration of these cells, which might be achieved by inhibiting the expression of its target VEGFA, and then downregulating the PI3K/Akt/CREB5 pathway.

## Data Availability

The original contributions presented in the study are included in the article/Supplementary Material, further inquiries can be directed to the corresponding authors.
