# Supplementary material for: Molecular Mechanism of Naringenin Against High-Glucose-Induced Vascular Smooth Muscle Cells Proliferation and Migration Based on Network Pharmacology and Transcriptomic Analyses
Source: Front Pharmacol. 2022 Jun 9;13:862709. doi: 10.3389/fphar.2022.862709 (PMC9219407; doi:10.3389/fphar.2022.862709)
Supplement: Supplementary file 2 [file Table1.DOCX]

Supplementary figures and captions

## We have 3 supplementary figures as follows.

## Supplementary Figures


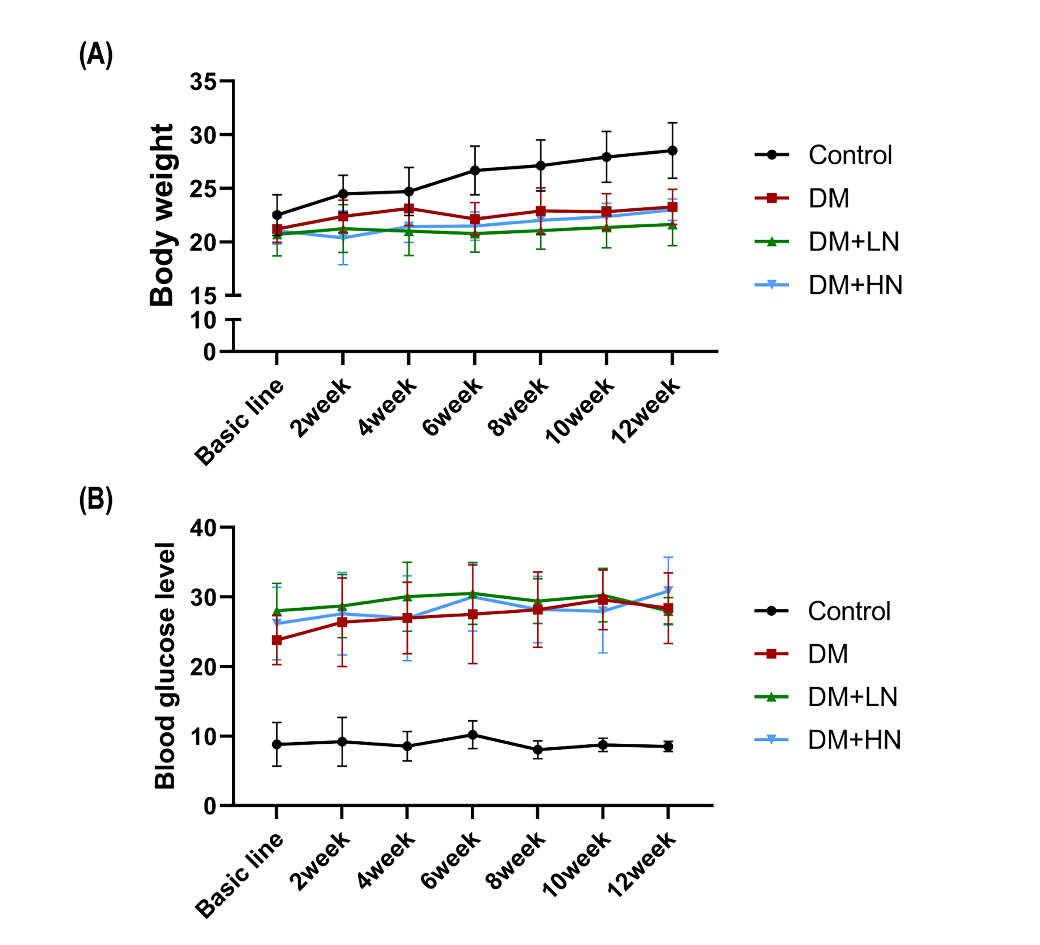


**Supplementary Fig.1.** variation of body weight and blood glucose in each group. (**A**) Compared with control group, the body weight of DM, DM+LN and DM+HN was significantly reduced. *p* < 0.05, but there were no significant differences among DM, DM+LN and DM+HN groups. (**B**) Compared with control group, the blood glucose of DM, DM+LN and DM+HN was significantly increased. *p* < 0.05, but there were no significant differences among DM, DM+LN and DM+HN groups.


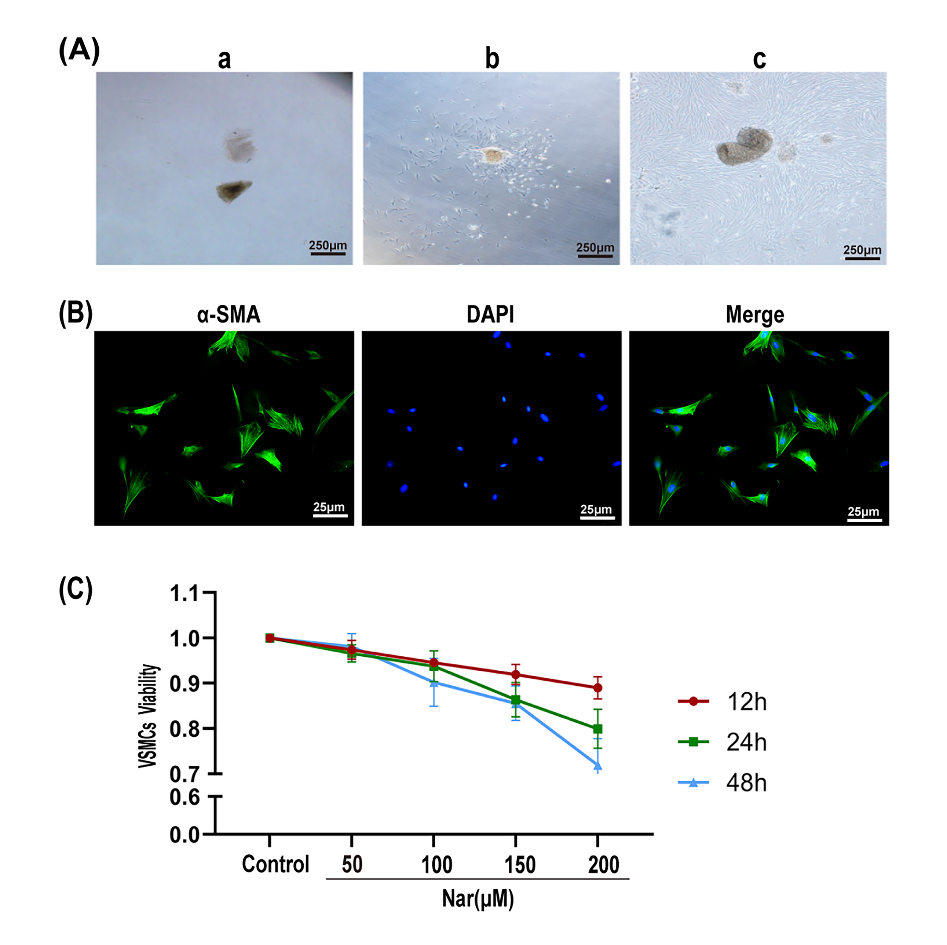


**Supplementary Fig.2.** (**A**) VSMCs were cultured by the tissue adherence method. (**B**) VSMC characterized by fluorescence microscopy. Expression of specific smooth muscle cell marker α-SMA was confirmed by green fluorescence. (**C**) CCK-8 evaluation of the effects of naringenin on VSMC proliferation in low glucose condition. No significant inhibition was found at the concentration of 50, 100, and 150 μM after diverse period of time treating with naringenin.


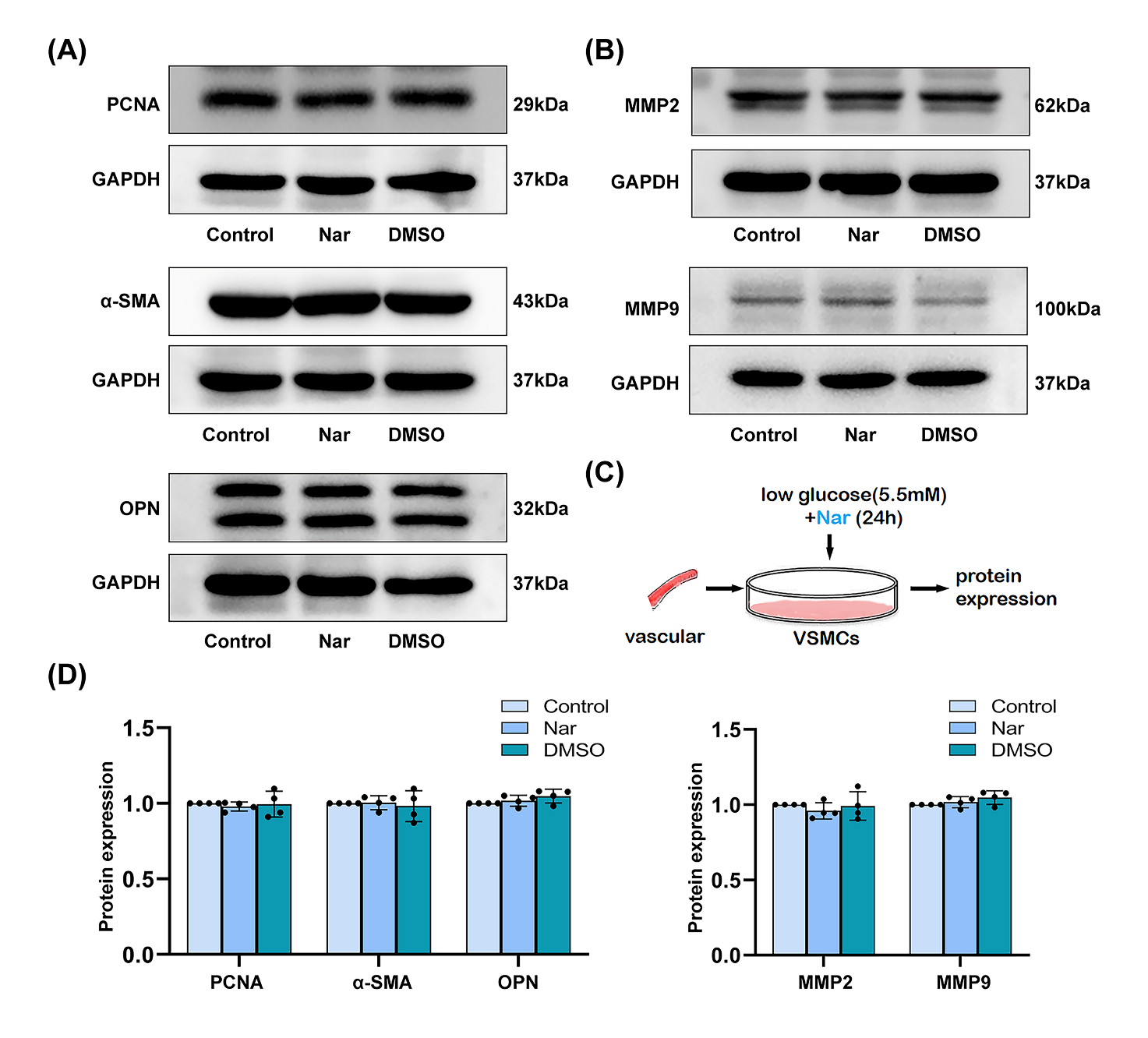


**Supplementary Fig.3.** Naringin and DMSO had no significant effect on VSMC proliferation, migration and surface differentiation. **(A)** Western blot detected the expression of PCNA, α-SMA and OPN. **(B)** Expression of MMP2 and MMP9. **(C)** Diagram of cell model. **(D)** Bar graph showed related protein expression analysis.
